# Managing Chronic Limb-Threatening Ischaemia in Patients at the Extremes of Older Age Requires a Patient-Focused Approach

**DOI:** 10.1007/s00268-022-06701-y

**Published:** 2022-08-18

**Authors:** Carina Cutmore, Sarah Aitken

**Affiliations:** Faculty of Medicine and Health, Vascular Surgery, Concord Repatriation General Hospital, The University of Sydney, Sydney, Australia

Older patients treated for chronic limb-threatening ischaemia (CLTI) have significantly higher mortality and adverse outcomes compared to patients who are younger. As the population of people aged 90 years and older is increasing rapidly, a considered approach to managing CLTI in patients at the extremes of older age is needed.

In this issue of the *World Journal of Surgery*, Dr Casajuana Urgell and colleagues share the results of their retrospective cohort study of 171 patients aged 90 years or older, who were diagnosed with CLTI [1]. They included nonagenarians in whom limb salvage was not considered (59, 35%), and those with potentially salvageable limbs who underwent either revascularisation (50, 29%), conservative treatment or minor amputation without revascularisation (57, 33%), or major amputation (5, 3%). By one year, more than half the overall cohort had died (95, 56%), with higher mortality in the group where limb salvage was not considered (49, 82%) compared to the group where limb salvage was considered (47, 42%). For the 50 patients who had revascularization, 33 patients had died at one year (30%) and two patients had major amputation. In contrast, of the 57 patients with potentially salvageable CLTI who were not revascularized, one patient proceeded to major amputation and 29 patients died (51%). Multivariable analysis demonstrated increasing age, congestive cardiac failure, dementia, being non-ambulant and pre-operative anaemia was predictive of reduced survival in patients with potentially salvageable limbs.

How do these findings by Casajuana Urgell et al. impact our current approaches to CLTI in patients of extreme older age? [1]. For many patients aged 90 years or older, their presentation with CLTI represents a terminal event for which neither amputation nor treatment appear to strongly influence. As Casajuana Urgell et al. demonstrate, whilst limb salvage is feasible in many patients of extreme age, long-term survival is poor. Revascularisation can improve quality of life and reduce pain for patients with CLTI, but these gains may be limited in non-ambulant patients [2]. For some older patients, amputation may be a preferable outcome to improve quality of life and health status [2]. This study by Casajuana Urgell et al. acts as a timely reminder that our approach to the care of older patients with CLTI needs to be adapted to their unique care needs [1].

The cumulative, and often interacting, effects of long-term multimorbidity and chronic care requirements make decision-making complex for older patients. Qualitative studies show that many older patients with CLTI value treatments that provide quality of life and function over potential survival or limb salvage gains [3]. Older patients want to be included in treatment decisions, but often wait for an explicit invitation to share their health care related priorities [3]. On the other hand, surgeons tend to predominantly focus on setting expectations and assessing risk, rather than specifically inviting patients to actively participate in decision-making, or only engage in shared decision-making when they are reluctant to operate [4]. The treatment priorities of patients can be overshadowed when mismatch occurs. There is increasing evidence that collaborative models of care between vascular surgeons and geriatricians can enhance shared decision-making and improve the care of older patients with vascular disease [5]. A more nuanced understanding of the progression of CLTI in patients of extreme older age can inform shared decision-making, and target treatment towards patient priorities.

A comprehensive evaluation of surgical risk and anticipated outcomes is critically important to inform CLTI management for older patients. Whilst Comprehensive Geriatric Assessment improves surgical outcomes, it is time consuming and requires specialist geriatrician input [5]. A more accessible way to assess surgical risk in older patients is to use validated frailty assessment tools. Frailty is increasingly recognized as predicting patients at risk of reduced survival and adverse CLTI outcomes. Non-ambulatory status and physical dependence act as frailty surrogates in Casajuana Urgell et al., predicting those patients unlikely to survive beyond one year [1]. Acknowledging the limitations of their single-centre, retrospective cohort study, Casajuana Urgell et al. report that premorbid function had an important influence on subsequent treatment decisions and outcomes [1]. The decision not to proceed with limb salvage in older patients is often driven by factors other than CLTI severity, such as severe dementia, concurrent terminal illness, or poor ambulatory function. The Global Vascular Guidelines provide an important guide for clinical decision-making in CLTI, but both the *Wound, Ischaemia and Foot Infection* (WIFI) and *Global Limb Anatomical Staging System* (GLASS) prognostic tools presume that an assessment overall patient risk and life expectancy has been completed [6]. Combining frailty assessment with other clinical decision-making tools, such as WIFI, may help distinguish which older patients will benefit most from CLTI revascularisation. Future modifications to CLTI decision-making algorithms should consider the inclusion of frailty assessment.

Opportunities for improvement also exist in how we report and measure CLTI treatment success for older patients. Metrics like amputation-free survival are important to compare and quantify variation in surgical outcomes, but often do not reflect what is important to older patients. The development of geriatric specific Quality Indicators (QIs), such as the American Geriatrics Society (AGS) Optimal Perioperative Management of the Geriatric Patient Best Practices Guidelines, present standards for measuring the quality of care provided older surgical patients. Whilst not specific to vascular surgery, many of the QI processes measure patient-centred outcomes like functional status, communication with carers and family, and cognitive screening. These process driven QI standards are potentially more reflective of the values of older patients than many reporting standards currently used in clinical trials and cohort studies of patients with CLTI [3]. Future research should both evaluate these geriatric specific QIs in vascular cohorts and focus on developing vascular specific QIs relevant to older patients.

Successful treatment of CLTI needs to be viewed differently in patients of extreme older age with an emphasis on quality of life and function, rather than just survival. Dr Casajuana Urgell and colleagues have added to this important conversation with their publication [1]. As the vascular community develop more nuanced clinical pathways for this vulnerable group of patients with CLTI, the focus must remain on the outcomes that matter most to our patients.
